# No Roadblocks: How Mobile Primary Care Navigates Barriers to Health Access

**DOI:** 10.3390/ijerph23070861

**Published:** 2026-06-30

**Authors:** Kayleigh E. Zinter, Ryan Burns, Ellen Pinnette, Trent B. Legare, Sheena C. McNeill, Alissa L. Sedelow, Jeremy Moseley, Jennifer Snow, Latoya Mallard Alexander, Renee M. Stakeman, Ashley B. Foster

**Affiliations:** Center for Clinical and Community Impact, Advocate Health, 1000 Blythe Boulevard, Charlotte, NC 28203, USA; ryan.burns@advocatehealth.org (R.B.); ellen.pinnette@advocatehealth.org (E.P.); trent.legare@advocatehealth.org (T.B.L.); sheena.mcneill@advocatehealth.org (S.C.M.); alissa.sedelow@advocatehealth.org (A.L.S.); michael.moseley@advocatehealth.org (J.M.); jennifer.snow@advocatehealth.org (J.S.);

**Keywords:** mobile health, mobile primary care, program evaluation, community health, health equity

## Abstract

**Highlights:**

**Public health relevance—How does this work relate to a public health issue?**

- The findings from this study indicate that mobile primary care can be utilized to address primary care access issues for medically underserved populations.

**Public health significance—Why is this work of significance to public health?**

- The findings from this study indicate that mobile primary care can be employed to connect people to long-term medical and social health services.

**Public health implications—What are the key implications or messages for practitioners, policy makers and/or researchers in public health?**

- This study indicates that healthcare organizations and mobile health program practitioners would benefit from employing multilingual practitioners to ensure immediate access to care for patients.
- Funding and support for mobile health and community-based healthcare programming should be inclusive of the relationship-building components of establishing community-based healthcare services.

**Abstract:**

Healthcare disparities are widespread across the U.S. Mobile health programming is designed to dismantle systemic healthcare barriers for medically underserved communities. The purpose of this study was to assess the effectiveness of two southeastern U.S. mobile primary care programs. Guided by the Social Determinants of Health framework, this descriptive study examines the programs’ patient populations to determine reach and patient medical data to ascertain patients’ connection to the medical system and long-term primary care. Data were collected via electronic health record (EHR) and analyzed in Power BI and R. Basic descriptive, crosstab and chi-square statistics were conducted to assess the patient population and explore patient healthcare engagement prior to and following their initial mobile primary care visit. The results indicate that mobile primary care programs are utilized by systemically underserved groups, with both program A and B’s patient population identifying primarily as Latin/Hispanic (69.6% and 41.3% respectively), uninsured (99.1% and 59.8%), and Spanish-speaking (64.4% and 42.3%). Additionally, each program demonstrates an ability to engage patients in long-term primary care utilization via repeat utilizers (65.5% and 25.9%). McNemar’s chi-square results show that participation in either of the mobile primary care programs has a statistically significant, non-random measurable effect on patient primary care enrollment. Differences in program structure and findings are explored, and recommendations for future practice, research, and policy are discussed.

## 1. Introduction

### 1.1. Healthcare

According to the 2024 Mirror, Mirror report, the U.S. ranked last overall among ten high-income countries in healthcare system performance [1]. United States healthcare was outperformed by other countries across metrics such as patient access to care, care outcomes, and service affordability [1]. While studies show the U.S consistently underperforms in key indicators such as life expectancy, preventable mortality, and maternal mortality [2,3].

Persistent disparities in healthcare access and outcomes across the U.S crucially impact overall health system performance. The Agency for Healthcare Research and Quality (AHRQ)’s Disparities Report consistently documents disparities in healthcare delivery related to factors such as race, income level, and social conditions [4]. These factors are notable across six priority areas: patient safety, person-centered care, care coordination, effective treatment, healthy living and care affordability. While the Affordable Care Act was designed to expand insurance coverage in the U.S. to improve healthcare access, approximately 26–27 million Americans remain uninsured as of 2024, accounting for approximately 8% of the U.S. population [5]. Coverage rates vary dramatically by race and ethnicity, with nearly one in four Hispanic adults aged 18–64 lacking health insurance, compared to lower rates among other demographic groups [5].

High costs of healthcare services and lack of insurance drive disparities in healthcare services and patient outcomes, and lead to under-utilization of preventative and primary care services [3,4,6]. As a result, many Americans are forced to later rely on emergency departments (EDs) and acute care settings to address conditions that could have been effectively and more efficiently managed earlier in primary care settings, contributing to avoidable hospitalizations, unaddressed health-related social needs, and poorer health outcomes [6,7].

### 1.2. Primary Care

Primary care is the provision of integrated, accessible healthcare services by clinicians who are accountable for addressing a large majority of personal healthcare needs, developing sustained partnerships with patients, and delivering care in the context of families and communities [8]. Primary care providers (PCPs) typically serve as the entry point for patients into the healthcare system [9].

A substantial body of evidence demonstrates that primary care plays a critical role in preventing illness, improving health outcomes, and supporting more efficient healthcare delivery. Additionally, rooted in a tradition of community-centered practice, primary care often prioritizes partnerships with patients, communities, and public health professionals to address social determinants of health (SDOH) [7]. Studies consistently show that a greater supply of primary care physicians is associated with improvements in population health [10,11]. Increasing the number of primary care physicians by just one per 10,000 individuals has been associated with an improvement in patient health outcomes, with improvements ranging from 0.66% to 10.8%, depending on the outcome measured [11]. Evidence demonstrates that a strong foundation of primary care yields better health outcomes overall, greater equity in healthcare access and outcomes, and lower per capita healthcare costs [6]. Primary care achieves these benefits in access and care quality by providing comprehensive services such as disease prevention, chronic disease management, and coordination with specialists [8].

International comparisons further highlight the importance of primary care. Countries that outperform the U.S in health outcomes tend to spend less overall on healthcare but invest significantly more in primary care and social services [12]. In the U.S, primary care accounts for more than 500 million visits annually, over half of all patient encounters, yet continues to receive a disproportionately small share of healthcare funding [12]. Shortcomings in primary care investment in the U.S disproportionately affect rural areas and communities of color that have historically faced barriers to accessing care [6].

### 1.3. Access Disparities

Access to primary care is not equal for all individuals. Health outcomes are often tied to where you live within a community, race/ethnicity, and poverty. The relationship between geography and health is frequently referred to as the “postcode lottery” and is observed globally [13]. In the U.S, individuals who reside in high-poverty zip codes struggle with lack of access to primary care while also contending with health outcomes negatively affected by income and geography [14]. For example, a baby born into poverty in the U.S can expect to live 10 to 15 fewer years than a child born into wealth [15]. Race is also a significant predictor of lifelong health, as studies nationwide demonstrate that intergenerational poverty, racism, and lack of healthcare access have had lasting effects on health and wellness within communities of color [16]. Access to primary care can help counterbalance these significant barriers to lifelong health. Patients whose physical, social, and behavioral needs are not well met via the healthcare system often accumulate high rates of ED visits and inpatient admissions that could have easily been prevented via utilization of primary care services or early intervention [17,18]. These patients are often dubbed “super-utilizers.” According to a literature review on this population, super utilizers often have a variety of comorbidities, and are more likely to be women, adults, experience housing instability, and have social health concerns [17].

### 1.4. Mobile Health Programming

Mobile health clinics in the U.S are dynamic, traveling healthcare clinics that can be implemented in a variety of formats. These mobile health clinics provide an opportunity to connect hard-to-reach patients to health and social care resources. Mobile health clinics are most often vehicles that have been customized or retrofitted to serve as a health clinic, traveling into communities to provide services to patients [19], but can also take the form of place-based rotating care options in a community. Mobile clinics have been found to successfully connect with underserved and vulnerable communities, overcome access barriers, build patient trust, improve rates of screenings and preventative care, and manage chronic diseases via unique and flexible modalities [19,20,21]. These clinics often serve high proportions of women [19,20], people of color [19,20], and uninsured or publicly insured patients [19]. Mobile health clinics can be designed to provide a variety of healthcare opportunities, including health education and vaccinations, preventative care, primary care services, specialty and social health care services etc. [19,22,23,24,25,26]. While often initially implemented as an opportunity to provide supplemental care services for patients and the medically underserved, according to a 2017 literature review, mobile health clinics are instrumental in providing continuous and encompassing care for patients and addressing health disparities [21]. In addition to reducing inequity and healthcare settings, extant research has also identified mobile health clinics to be particularly useful for adults with uncontrolled chronic conditions such as hypertension and diabetes [20,27] and an opportunity to reduce ED utilization and associated costs to patients and healthcare systems [27].

### 1.5. Mobile Primary Care

Mobile health clinics span a multitude of service areas, one of which is *primary care provision*. Mobile primary care services are a promising, innovative, ambulatory-forward alternative to traditional brick and mortar primary care facilities, and take a variety of formats (e.g., mobile vans or buses, rotating place-based clinics, community care clinics, school-based health clinics etc.). Mobile primary care patient population demographics mirror those of mobile health patients broadly, often serving a higher percentage of women, people of color, and under- or uninsured patients [19,22,28]. These clinics connect hard-to-reach patients to medical services, improve chronic disease management, and promote wellness in their target communities. For mobile health clinics to be successful, extant research has demonstrated the necessity of community buy-in, stressing the importance of community perceptions of mobile primary care clinics, relationship building, trust and collaboration for maximum return on investment and utilization [28,29,30]. In addition to increasing access to primary care services for medically underserved populations, mobile primary care has also been identified as a potentially cost-saving alternative to brick-and-mortar facilities. Attipoe-Dorcoo and colleagues’ [31] study in the Southern U.S. found that the cost of operations of mobile primary care clinics is often less than that of fixed clinics, with the majority of costs going to staff and labor, with limited overhead-associated costs. Given their ability to reach a variety of patients, adapt in modality to their surroundings, and potential for cost savings, mobile primary care clinics are, in some instances, an important and perhaps necessary alternative to brick-and-mortar facilities with limited reach and high associated costs.

### 1.6. Current Study

The mobile health programs across Advocate Health are groundbreaking initiatives designed to dismantle systemic barriers to healthcare for underserved communities. Advocate Health is one of the largest vertically integrated health systems in the U.S. The North Carolina and Georgia (NC/GA) division of Advocate Health is home to the two longest-operating mobile primary care programs across the larger Advocate system. The current study assesses the efficacy of these two mobile primary care programs, located in two different metropolitan areas and their surrounding counties across western North Carolina.

Through data-driven strategies and strong community partnerships, these mobile primary care programs deliver targeted, culturally competent care via mobile health clinical primary care provision. These fully equipped clinics provide diagnostic services, prescriptions, vaccinations, patient education, and acute care, meeting patients where they are to address critical health needs. Rooted in the Social Determinants of Health (SDOH) framework and using the Center for Disease Control and Prevention’s (CDC) Social Vulnerability Index [32], the programs have identified high-risk geographic areas where residents face substantial barriers to care, including transportation challenges, disabilities, inflexible work schedules and lack of insurance, which results in delayed treatment and preventable medical issues. The SDOH framework aids in assessing the extent to which these mobile primary care programs are reaching individuals with the greatest unmet need [33].

### 1.7. Research Questions and Hypotheses

This retrospective study was conducted to assess the impact of mobile primary care programming on healthcare access. This study had two primary research questions: (1) Who is the population the mobile primary care programs serve? and (2) How do these mobile primary care programs impact healthcare utilization in their patient population? Specifically, is there any change in patient utilization and connection to primary care services?

These programs were developed to improve access to healthcare services for populations that have historically been medically underserved. As such, the expectation is that, for both mobile primary care programs, the patient population will demographically reflect medically underserved groups and connection to healthcare services will increase as a result of program utilization.

## 2. Materials and Methods

### 2.1. Study Setting

#### 2.1.1. Mobile Primary Care “Program A” Overview

Advocate Health’s first mobile primary care program, Program A, was initially conceived as a free clinic in collaboration with an East Winston-Salem church beginning in 2015. Initially the goal of the free clinic was to provide compassionate and holistic care in a neighborhood that had faced numerous barriers to accessing appropriate medical care. This clinic illuminated the need for a healthcare model that could serve multiple neighborhoods and populations. In late 2019, based on community feedback and listening sessions, the free clinic program was revised to operate as a walk-in urgent-care-on-wheels or a mobile health program. Over time, continued feedback from community members highlighted the need for consistent providers and a medical home for patients. To better address community needs, in January 2021, the program’s focus shifted to providing primary care. The program has continued to evolve and presently offers primary care services embedded across four rotating neighborhood care sites in the focal community weekly.

All patients served by Program A lack health insurance and access to affordable primary care. Operating as a free and charitable clinic, the mobile primary care program does not bill patients for services. The clinic is fully equipped and has partnerships within the local medical system that provide free lab tests and supplies.

#### 2.1.2. Mobile Primary Care “Program B” Overview

The healthcare system’s second mobile primary care program, *Program B*, launched in Charlotte in September 2022 as part of a strategy to expand access to care and improve health outcomes in underserved areas. Building on the success of earlier mobile COVID-19 efforts in Charlotte, Program B was designed to bring comprehensive clinical services directly into underserved communities across the Greater Charlotte region, centering on primary care services.

Program B began with three, but currently operates two fully equipped mobile primary care clinics on wheels. These clinics offer preventive care, chronic disease management, minor injury treatment, pregnancy and sexually transmitted infection (STI) testing, and referrals to specialty and social services. Together, the two units rotate through over 10 community sites across the greater Charlotte metropolitan area, including churches, schools, and community centers, and have reached patients from more than 300 ZIP codes since program inception.

### 2.2. Participants

Across both mobile primary care programs A and B, a total of 4365 patients were served from the mobile primary care programs’ inception (January 2021 and September 2022 respectively) through December 2025. Most patients served across both programs were adults aged 26–64 (*n* = 2902; 66.5%), female (*n* = 2501; 57.3%), uninsured (*n* = 3499; 89.3%), Hispanic/Latinx (*n* = 2492; 57.1%), and reported Spanish as their preferred language (*n* = 2385; 54.6%). For details across all programs, please see the patient demographic information table (Table 1) below.

### 2.3. Materials and Procedures

All patients who utilize Advocate Health medical services have their patient and health information stored via electronic health record (EHR). Following receipt of Institutional Review Board (IRB) exemption, secondary data analysis was conducted utilizing de-identified mobile health patient EHR data. Patient data were extracted for existing mobile health patients from program start through December 2025.

### 2.4. Measures

Electronic health record data of interest for this study include the following categorical and continuous variables: mobile primary care program participation (i.e., is the patient a Program A or Program B patient?); patient demographic information (i.e., race/ethnicity, sex, age, language, payor status, postal code); and healthcare utilization and long-term connection to healthcare services. Healthcare utilization and connection variables include PCP attribution pre-index visit (i.e., whether or not the patient had any recorded organizational primary care provider prior to their first program visit), PCP attribution post-index visit (i.e., whether or not the patient had any recorded organizational primary care provider following to their first program visit), specialty care referrals (i.e., did the patient receive a referral to a specialty medical service), and number of primary care visits pre- and post-index visit (number of organizational primary care primary care visits a patient had prior to and following their first mobile program visit; see Appendix B for full list of measures).

### 2.5. Analysis

For this study, basic descriptive statistics (i.e., frequencies) and crosstabulation (crosstab) analyses were conducted in Microsoft Power BI and R. Crosstabs are used to summarize categorical data in tables using frequencies to test hypotheses associated with categorical variables [34]. In this study, categorical variables of interest included mobile primary care patients, insurance status, race/ethnicity, primary care attribution, chronic disease diagnosis, and specialty care connection. In this study, crosstabs were used to identify similarities and differences between each program, as well as identify differences in healthcare utilization pre- and post-index mobile primary care visit (i.e., patients’ first visit with the mobile health program).

In addition to descriptive statistics, a McNemar’s chi-square was also conducted [35]. The McNemar’s test is a non-parametric statistical test utilized to determine if a statistically significant difference exists between proportions resulting from paired data [35]. This test is ideal for “before-and-after” intervention designs, where subjects serve as their own controls, as in this study [35].

## 3. Results

### 3.1. Mobile Primary Care Programs

#### 3.1.1. Access and Patient Population

##### Patient Demographics

Program A served patients aged 10 and older (*R* = 10–100 years old), with a mean age of 46 years. Most patients were Spanish-speaking (*n* = 1573, 64.4%), with 70% stating a non-English preferred language. Further, nearly 100% of patients (*n* = 2423) were uninsured. See Table 1 for a comprehensive view of Program A’s patient demographic information.

Program B served clients aged 2–101 years, the majority of whom were adults, with a mean age of 36. Most patients were Hispanic or Latino (*n* = 794, 41%) and had a preferred language of English (*n* = 976, 51%). Approximately 60% were uninsured at the time of their appointment. See Table 1 for a comprehensive view of Program B’s patient demographic information.

##### Long-Term Connection to Healthcare Services

A McNemar’s chi-square test [35] was conducted to assess whether program participation was associated with a change in primary care attribution in patients eligible for 12-month pre-post analyses. The results indicate that the mobile programs’ intervention has a statistically significant, measurable, non-random relationship with attribution to primary care in the 12 months following patients’ first mobile program visit, compared to their primary care attribution in the year prior (McNemar’s *χ*^2^ (*n* = 3238) = 1407.4, *p*-value < 0.001). Specifically, there is a statistically significant difference in the proportion of patients who enroll in primary care after exposure to mobile primary care program services. Given that the count of patients who switched from non-attribution to attribution (*n* = 1474) is far higher than the count of patients who switched from attributed to not attributed to primary care (*n* = 22), we can infer that program participation is successful in connecting patients to a primary care provider.

### 3.2. Program A

#### 3.2.1. Program Reach and Growth

Since Program A began operating in January of 2021, providers have served 2444 unique patients, with a total of 8356 visits. Program A patients represent 937 different U.S. postal codes across eight different states. The majority of patients are from North Carolina (*n* = 2432 patients, 927 postal codes). The states of South Carolina, Florida, Georgia, New York, Tennessee, Texas, and Washington are also represented in the program’s patient population. Program A has generally seen stable utilization after a drop-off following the program’s first year. In addition to typical primary care services, Program A also served as a COVID-19 vaccination clinic in 2021.

#### 3.2.2. Long-Term Connection to Healthcare Services

##### Primary Care Utilization

Of patients who were eligible for one year post-index visit analyses (*n* = 2146), a majority 72% (*n* = 1546) had *not* been to or enrolled in primary care (i.e., primary care attribution) prior to their first mobile primary care visit, while 27% (*n* = 583) had no primary care attribution data reported, and less than 1% (*n* = 17) were enrolled with a PCP. See Table 2 for complete primary care and mobile primary care program utilization information. In the 12 months following their index visit, 446 patients (21%) were attributed to primary care, and 79.1% (*n* = 1699) were not attributed to primary care, with less than 1% having no primary care attribution data to report.

Patients do not have to enroll with a particular PCP to have a primary care visit (see Appendix A for a complete list of primary care services). Of *Program A* patients eligible for one year post-index visit analyses (*n* = 2146), 98.6% had zero PCP visits prior to their first index visit, with less than 2% of mobile primary care patients having any number of PCP visits in the year prior to their first index visit. Following their first index visit, 84.0% of patients had no PCP visits, 5.6% had only one visit, and 10.4% had two or more PCP visits.

##### Repeat Program Patients

As of 31 December 2025, 2444 unique mobile primary care patients have been served by *Program A*. Of those patients, 65.5% (*n* = 1601) were repeat mobile primary care program utilizers, while 34.5% (*n* = 843) of patients had only one visit. Of the 1601 repeat program utilizers, the number of repeat visits ranged from 2 to 47 visits per patient (*M* = 4.82 visits, *Mdn* = 3 visits).

##### Referrals and Specialty Care Connection

Primary care is just one aspect of the healthcare system, and often patients require additional healthcare services. Mobile primary care programming can act as a patient’s entry point to the broader healthcare system. For *Program A* patients, 1011 referrals were made by the provider during their mobile primary care visits for additional services. Of these referrals, 54.5% (*n* = 551) were to specialty care providers (gastroenterology, dermatology, oncology, etc.), 18.7% (*n* = 189) were for further diagnostic testing, 15.5% (*n* = 157) were social care referrals (i.e., orders placed in the EHR for Community Health Worker support), 8.4% (*n* = 85) were ancillary care referrals (i.e., supportive, non-physician services that assist in the diagnosis, treatment, and management of patient conditions), 2.1% (*n* = 22) were unspecified referrals, and less than 1% were requested consultations.

While patients can be referred to other providers or further services, it is not guaranteed that patients will connect with these additional support services. When examining the referral status of the 1011 referrals made, 62.3% (*n* = 630) were considered completed. Of the remaining referrals, 21.5% (*n* = 217) were approved/authorized and open, or not considered complete; 15.5% (*n* = 157) were pending review; and less than 1% were cancelled.

### 3.3. Program B

#### 3.3.1. Program Reach and Growth

Since *Program B* began operating in September 2022, providers have served 1921 unique patients with a total of 2772 visits to the mobile program. Program B patients were from 361 different postal codes across seven different states. Most patients were from North Carolina (*n* = 1895 patients, 343 postal codes). The states of South Carolina, Ohio, Virginia, New York, Wisconsin, and Minnesota were also represented in the program’s patient population. Program B continued to see a large percentage of growth in utilization and reach year over year, indicating it has not yet reached peak service provision. It should be noted that the rate of increase in the number of unique patients has dropped each year, indicating that the program may be nearing capacity for the number of unique patients able to be seen each year.

#### 3.3.2. Long-Term Connection to Healthcare Services

##### Primary Care Utilization

Of patients who were eligible for one year post-index visit analyses (*n* = 1230), the majority of patients, 70.6% (*n* = 868), had *not* been to or enrolled in primary care prior to their first mobile primary care visit, while 29.4% (*n* = 362) reported pre-existing primary care attribution. In the 12 months following their index visit, only 16.2% (*n* = 199) of patients were *not* attributed to primary care, while 1031 (83.8%) were enrolled in primary care in the 12 months following their first mobile primary care visit. Less than 1% of patients had no data for primary care attribution in the 12 months prior to or following their first index visit (See Table 3).

Of *Program B* patients eligible for one-year post-index visit analyses (*n* = 1230), 74.0% had zero PCP visits prior to their index visit, 8.8% had one visit, while 17.2% had two or more visits in the time leading up to their first mobile primary care visit. Following their index visit, only 47.7% of patients had no PCP visits, while 15.9% had one visit, and 36.3% had two or more PCP visits in the year following their initial *Program B* visit.

##### Repeat Patients

Of the 1921 MPC patients served by *Program B*, 25.9% (*n* = 497) were repeat program utilizers. In addition, 74% (*n* = 1424) of mobile primary care patients had only one visit. Of the 497 repeat program utilizers, the number of repeat visits ranged from 2 to 12 visits per patient (*M* = 3.03 visits, *Mdn* = 2 visits).

##### Referrals and Specialty Care

Primary care is just one aspect of the healthcare system, and often patients require additional healthcare services. Mobile primary care programming can act as a patient’s entry point to the broader healthcare system. For *Program B* patients, 1475 referrals were made by providers during mobile primary care visits for additional services. Of these referrals, 52.1% (*n* = 768) were to specialty care providers (gastroenterology, dermatology, oncology, etc.), 26.6% (*n* = 393) were for further diagnostic testing, 12.2% (*n* = 180) were social care referrals, and 9.1% (*n* = 180) were ancillary care.

While patients can be referred to other providers or further services, it is not guaranteed that patients will connect with these additional support services. When examining the referral status of the 1475 referrals made, 67.6% (*n* = 997) were considered completed. Of the remaining referrals, 26.8% (*n* = 395) were approved or authorized, but not considered complete; 2.0% (*n* = 30) were pending review; and 3.6% (*n* =53) were cancelled or rejected.

## 4. Discussion

### 4.1. Overview

When looking at the programs together, 4365 unique patients were seen, spanning over 11,000 visits to these mobile primary care services since 2021. Data from this study supports existing the findings that mobile primary care program patient populations are mostly women, uninsured, people of color, and patients with uncontrolled chronic conditions such as diabetes or hypertension, and adds to the current body of literature by identifying patients served by these programs as predominantly non-English speaking (60.9%), specifically Latino/Hispanic, and as patients who had not been seen or enrolled in primary care in the year prior to their first mobile primary care visit [18,21,27]. The study results also indicate that mobile health patients hail from a wide range of locations geographically, even beyond the areas where the clinics travel. These results align with the SDOH theoretical framework, as both programs consistently reached high-risk geographic areas where residents face substantial barriers to care, including transportation challenges. The specialty care referral findings support the idea that both programs address social and medical needs beyond a single visit.

Further, this study examines how patients engage with the program and medical system following their initial visit. Most extant mobile health research is focused on cost of operations [30], patient population [18,21,27], community engagement [27,28,29], types of services [18,21,22,23,24,25], and implementation modalities [18,20,28]. These data also provide insight into how patients use mobile health clinics and other medical services available to them pre- and post-index visit across the two program modalities. Both Program A and Program B saw an increase in primary care attribution of their patients following their mobile index visit, as well as an increase in PCP visits from pre to post. Additionally, both programs referred patients to other providers and programs for additional care and services.

### 4.2. Program A vs. Program B

While the two programs share similarities across patient demographics, reach, and referrals, there were also key differences in patient utilization of the mobile programs. For example, *Program A* patients were primarily repeat utilizers (65.5%), while *Program B* patients were repeat utilizers at less than half that rate, with only 25.9% of patients engaging with mobile primary care services multiple times during the study time frame. Conversely, the rate at which *Program B* patients attributed to and utilized further primary and specialty care services across the healthcare system increased exponentially from pre to post (an increase of 29.4% to 83.8% for patients for primary care attribution, and an increase of 26% to 52.3% for patients with primary care visits). While *Program A* saw changes in both categories, these did not occur in such high percentages, and there was a higher degree of missing data.

These differences may be due to the differences in mobile primary care implementation between *Programs A* and *B*. The majority of patients across both programs were uninsured. As *Program A* operates as a free and charitable clinic, it is likely that patients, particularly those who were uninsured, would utilize the program repeatedly for their healthcare needs. Conversely, for *Program B*, while there were options for patients to receive free or reduced-cost care that the program implemented and shared with its patients, the program did bill for services. The paperwork and process to determine whether patients qualified for financial assistance were time-consuming, and when patients went through this process, typically with staff, it was explained to them that once patients qualified for financial assistance, they were eligible for free healthcare services across the entire focal healthcare system. As such, patients who qualified had the opportunity to utilize a multitude of health services outside of those provided exclusively by the mobile primary care program. This may have been a feature that was particularly appealing to patients who had previously not received medical care during the time period before their first mobile primary care visit. Additionally, the differing setups of the two programs may have also impacted how the programs were utilized. *Program A* provided services across four rotating, embedded community-based sites, while *Program B* provided services across more community-based sites, operating out of a mobile care bus that parked at their focal sites rather than an embedded location. Lastly, *Program A* being in operation longer provided *Program A* utilizers with more opportunities for repeat patient visits *than Program B*, and some of these differences in repeat patient utilization may be a product of program longevity rather than operational and implementation differences.

### 4.3. Impact of Program Utilization

As medically underserved patients typically end up being “super-utilizers” of hospital systems, and the mobile health patient population is similar to that of super-utilizers (i.e., women, adults, low-income earners and/or uninsured patients, and often face multiple, overlapping barriers to care [17,18]), it is promising that Program A and B patients end up being repeat program patients, and/or connect to further healthcare services once they have been established as mobile primary care patients. This may have long-term implications for the super-utilization of EDs and hospitals in focal geographic regions. Viewed through the SDOH framework, these findings suggest that mobile primary care may help mitigate access-related consequences of overlapping structural barriers such as lack of insurance, language-related barriers, transportation challenges, and difficulty navigating the healthcare system [17].

### 4.4. Limitations

This study is not without limitations. As a comparative observational study, causality of patient healthcare utilization is unable to be determined. This study is quantitative, and important context around the “why” behind certain outcomes is not available using exclusively quantitative methodologies. Further, both programs are based in the state of North Carolina, and thus, generalizability across mobile health programs more broadly is not recommended. Lastly, while this study does look at some pre/post effects of mobile primary care programming, due to the limited time frames (12 months pre and 12 months post), we opted not to explore chronic disease management outcomes, as long-term changes and differences in health status may take longer to achieve. Once the programs have been in operation for a longer period, these are the data we plan to revisit.

### 4.5. Implications

#### 4.5.1. Research

While this study provides a solid foundation to further understand how mobile primary care programs are utilized by patients and indicates that they positively influence access to healthcare for medically underserved populations, additional quantitative research on the topic is needed. Future research should include not only descriptive statistics like in this study but also tests of statistical significance and robust modeling, inclusive of predictor and confounding variables, to better understand the effect of mobile primary care programming on patient connection to healthcare programming and long-term service utilization. Future quantitative analyses that are inclusive of patient health outcomes, such as changes in key chronic disease markers and specialty care referral follow-through, as well as exploration of the effect of mobile primary care specifically on emergency health services utilization, are also important next steps in this line of inquiry. While previous research has found that mobile health programs may be effective tools for reducing ED utilization in certain populations [18,27,36], this has not been explored specifically within the communities served by these two mobile programs. Beyond further quantitative analyses, future research should also utilize mixed and qualitative methodologies to better understand program impact and patient experience. It is also important to note that extant research has discussed how utilization may differ based on geographic location and degree of urbanicity. As both programs serve patients from a wide range of zip codes and regions, it may be useful to explore differences in utilization based on geographic context in future studies.

#### 4.5.2. Practice

The findings from this study have several implications for mobile primary care practice. First, there is the necessity to prioritize language services to promote access, as the majority of patients served had a non-English preferred language. Accurate and effective translation services allow mobile programs to immediately serve diverse patient populations. Committing to providing comprehensive language services to patients at organizational and programmatic levels allows mobile programs to successfully reach patients in the U.S. who do not speak English as their primary language. While the focal medical system employs virtual interpreters staff can contact as needed, given that the preferred language for the majority of the mobile health patients is Spanish, the programs may consider during hiring of staff preference for bilingual or native Spanish speakers as part of the care team, or hiring interpreters designated specifically to serve the mobile primary care programs.

Extant research lauds the need for community buy-in, trust building, and the importance of community perception for the success of mobile health programming [28,29,30]. Upon examining both programs’ utilization metrics year over year, study data may also reflect the necessity of community building and buy-in for successful implementation of mobile health programming. *Program A*, which was developed in collaboration with a local church and operated as a free clinic, then mobile urgent care, before transitioning to mobile primary care services, had been active in and responsive to the needs of the community for six years before the mobile primary care iteration of service provision was launched. From the beginning of the study, this program was operating and providing services for patients at capacity, and the data reflects little fluctuation in visit volume from 2021 to 2025. Alternatively, *Program B* was launched by the focal healthcare system as a strategy to expand primary care access, utilizing SVI data to identify target communities. This program engaged in relationship-building activities during, rather than prior to, implementation. Initially, program utilization by the target communities was quite low. However, over time, utilization steadily increased. This may be, in part, a product of trust and relationship-building efforts, and patients learning about care provision on the mobile units via word of mouth. As further mobile primary care programs are implemented, it is important to consider the focal community to ensure service provision is meeting the needs of the target population, not only while the program is operational, but also before. These activities may be instrumental for determining program success.

#### 4.5.3. Policy

The success of mobile health clinics relies on supportive policy at all levels: organizational, local, state, and federal government. During times of changing priorities in federal healthcare policy, healthcare organizational policy may help secure affordable and accessible care for medically underserved patients via mobile health programs [37]. Organizational commitment to free mobile health programs can mitigate patients’ loss of healthcare access due to changes in Medicare and Medicaid eligibility. Organizational policy committing all care in a mobile health program to be free also reduces time-intensive and burdensome paperwork that patients unable to pay for services would typically be required to complete to receive organizational financial assistance. Removing burdensome administrative operations reduces time spent on paperwork and allows mobile health care teams to use their time more exclusively for patient care.

Mobile health is uniquely positioned to serve physically hard-to-reach rural populations. Government policy that seeks to promote rural healthcare access or improve chronic disease outcomes among rural populations should incorporate explicit support for the development and implementation of mobile health programs. Olson and colleagues [38] found that U.S. migrant farmworkers experience extensive access barriers to health care and chronic disease management. As mobile health clinics are particularly adept at serving vulnerable populations, implementation of mobile primary care and mobile health services may be a key prong in reaching this medically underserved community.

Policy designed to support the development and relationship-building period of community-based programming is also a key component to the success of a rural health improvement strategy. The data from this study emphasizes that community buy-in is a critical aspect of mobile health program success. Supportive policy allows organizations to allocate greater time to building community relationships, which may result in better reach within the community. Conversely, it is crucial to note that non-healthcare policy may also affect mobile programs’ ability to reach target populations. Increasingly strict federal immigration enforcement in the U.S. may discourage target populations from seeking care with mobile health programs, limiting programmatic reach. This was highlighted as a potential concern across both programs, during months and years when mobile primary care communities were targeted by the Department of Homeland Security and Immigration and Customs Enforcement, and program utilization decreased. When governments prioritize achieving a reduction in healthcare access disparities and improved chronic disease management, policy must broadly support the connection of medically underserved populations with healthcare, such as through mobile health programs. The current study contributes to evidence that mobile health programs successfully reach medically underserved populations, but further research is necessary to understand the relationship between immigration policy and mobile health program reach.

## 5. Conclusions

Race and poverty negatively affect health. People with significant access barriers to services such as geographic and transportation-related barriers, language-related barriers, disabilities, inability to take time off work to attend appointments, difficulty navigating the healthcare system, lack of insurance coverage, or other cost concerns or access issues are often medically underserved, resulting in poor health over the lifetime. These barriers work together multiplicatively to impede access to health care. Patients without primary care arrive at the ED with acute medical needs that are often preventable. Innovative solutions are needed to provide underserved residents with affordable, accessible healthcare that will improve overall health and wellness outcomes. Mobile primary care is one such innovative solution. Mobile primary care programs can be utilized to target service provision in medically underserved communities, thus promoting healthcare access and increasing the ability of healthcare systems to reach these groups prior to medical needs requiring acute services.

## Figures and Tables

**Table 1:** Mobile primary care (MPC) patient demographic information.

Patient Demographics	MPC		Program A		Program B	
	*n*	*%*	*n*	*%*	*n*	*%*
Patient race/ethnicity						
Hispanic/Latino	2492	57.09	1698	69.48	794	41.33
Black/African American	754	17.27	261	10.68	493	25.66
White/Caucasian	604	13.84	215	8.80	389	20.25
Bi/Multi-Racial	302	6.92	119	4.87	183	9.53
Not Specified	152	3.48	126	5.16	26	1.35
Other	61	1.39	25	1.02	36	1.87
Patient sex						
Female	2501	57.30	1428	58.43	1073	55.86
Male	1864	42.70	1016	41.57	848	44.14
Patient age in years						
2–17	620	14.20	56	2.29	564	29.36
18–25	334	7.65	210	8.59	124	6.46
26–64	2902	66.48	1879	76.88	1023	53.25
65+	509	11.66	299	12.23	210	10.93
Patient preferred language						
Spanish	2385	54.64	1573	64.36	810	42.17
English	1705	39.06	729	29.83	978	50.91
Portuguese					94	4.89
Other ^a^	275	6.30	142	5.81	39	2.03
Patient insurance/payor status						
Self-pay	3499	89.29	2423	99.14	1076	59.78
Medicaid	413	4.41			392	16.52
Commercial	383	4.47			367	17.10
Medicare	138	1.56			137	6.24
Other ^a^	20	0.27	21	.86	10	0.36

Note. MPC *N* = 4365, Program A *n* = 2444, Program B *n* = 1921. ^a^ Please note that, for some “demographic” categorizations (i.e., preferred language and payor status), if the percentage of patients was under 1% for a program, existing demographic options were consolidated into the “Other” category in this table to avoid possible identifiability.

**Table 2:** Program A patient primary care connection pre- and post-index visit.

Measure	Pre-Index			Post-Index		
	*n*	*%*	*Valid%*	*n*	*%*	*Valid%*
PCP attribution						
Yes	17	0.79	1.09	446	20.78	20.79
No	1546	72.04	98.91	1699	79.17	79.21
No data	583	27.17	-	1	0.05	-
Number of patients with PCP visits						
0 visits	2116	98.60	-	1803	84.02	-
1 visit	16	0.75	-	119	5.55	-
2 or more visits	14	0.65	-	224	10.44	-
MPC patient number of visits ^a^						
1	-	-	-	843	34.49	-
2 or more	-	-	-	1601	65.51	-

Note. Pre-index *n* = 2146, post-index *n* = 2146. Mobile primary care visits do not count toward patients’ PCP visit numbers. ^a^ MPC *N* = 2444, *M* = 4.82, *Mdn* = 3.

**Table 3:** Program B patient primary care connection pre- and post-index visit.

Measure	Pre-Index		Post-Index	
	*n*	*%*	*n*	*%*
PCP attribution				
Yes	362	29.43	1031	83.82
No	868	70.57	199	16.18
Number of patients with PCP visits				
0 visits	910	73.98	587	47.72
1 visit	108	8.78	196	15.93
2 or more visits	212	17.24	447	36.34
Mobile primary care number of visits ^a^				
1	-	-	1424	74.13
2 or more	-	-	497	25.87

Note. Pre-index *n* = 1230, post-index *n* = 1230. Mobile primary care visits do not count toward patients’ PCP visit numbers. ^a^ MPC *N* = 1921, *M* = 3.03, *Mdn* = 2.

## Data Availability

The data presented in this study are available on request from the corresponding author on a case-by-case basis. The data contained in this study would need to be cleaned and deidentified, and a data-sharing agreement would need to be drafted and processed prior to any release.

## Appendix A. Primary Care Visit Types

A primary care encounter is a closed, outpatient encounter associated with any one of the following visit types and department specialties.

**Table A1:** Primary care visit types.

Office Visit	Worker’s Comp	OB Check
Hospital	Pre-Admit	Postpartum Visit
Immunization	Walk In	Research Oncology
Research Encounter	Group Visit	Nutrition
Confidential	Nursing Home Visit	Initial Consult
Nurse Only	Home/Group Home Visit	Clinical Support
Occ Health	First OB Visit	Routine Prenatal
Anti-Coag	Follow-Up	Radiation Oncology
Walk-In	Pediatric	Procedure Visit
Evaluation	Transplant Surgery	Surgical Consult
Initial Prenatal	Ophthalmology	Case Management
Employee Health	Alcohol/Drug Abuse	Home Care Visit
Behavioral Health	Telemedicine	

**Table A2:** Primary care department specialties.

Family Medicine	Gynecology	Primary Care
General Internal Medicine	Obstetrics	Urgent Care
Geriatric Medicine	Obstetrics and Gynecology	Adolescent Medicine
Gerontology	Pediatrics	

## Appendix B

**Table A3:** Study measures.

Variable	Definition	Variable Type
Patient Demographics		
Race/Ethnicity	Combination of race and ethnicity variables; if no ethnicity is selected, race selection is used	Categorical
Sex	Sex assigned at birth as determined by a medical or birthing professional	Categorical
Age	Patient age in years	Continuous
Payor Status	Patient insurance information at first visit	Categorical
Postal Code	Patient postal code at the time of the index visit	Continuous
Preferred Language	Patient preferred language to use during the visit	Categorical
Patient Healthcare Utilization		
PCP Attribution (Pre-Index)	Whether or not the patient had a recorded organizational primary care provider in the two years prior to their index visit	Categorical
PCP Attribution (Post-Index)	Whether or not the patient had a recorded organizational primary care provider in the 12 months following their index visit	Categorical
PCP Visits (Pre-Index)	The number of primary care visits a patient had in the 12 months prior to their index visit	Continuous
PCP Visits (Post-Index)	The number of primary care visits a patient had in the 12 months following to their index visit	Continuous
Total Visits	Number of patient visits to the mobile primary care program	Continuous
Index Visit	Date of first visit to the mobile program	Continuous
Referral Type	Referral categorization of the referral made during the mobile health appointment	Categorical
Referral Status	What is the status of the referral made during the mobile health appointment?	Categorical
